# Kostmann Syndrome With Neurological Abnormalities: A Case Report and Literature Review

**DOI:** 10.3389/fped.2020.586859

**Published:** 2020-12-14

**Authors:** Baiyu Lyu, Wei Lyu, Xiaoying Zhang

**Affiliations:** Department of Pediatrics, Shanghai Ninth People's Hospital, Shanghai Jiaotong University School of Medicine, Shanghai, China

**Keywords:** Kostmann syndrome, HAX1 gene, neurologic manifestations, severe congenital neutropenia, epilepsy

## Abstract

**Background:** Severe congenital neutropenia (SCN), also known as Kostmann syndrome, is a rare heterogeneous group of diseases characterized by arrested neutrophil maturation in the bone marrow.

**Case Presentation:** We report a case of Kostmann syndrome and review previously reported SCN cases with neurological abnormalities. A 10-year-old boy had a history of recurrent, once a month, infection starting at 6 months of age. He had neutropenia for more than 9 years, as well as intellectual disability. He was homozygous for the exon 3 c.430dupG mutation of the HAX1 gene NM-006118. After treatment of antibiotics and G-CSF, his symtoms were relieved and was 3 months free of infection. The search revealed 29 articles related to Kostmann syndrome caused by HAX1 gene mutation; they were screened, and the main clinical features of 13 cases of Kostmann syndrome with neurological abnormalities were summarized and analyzed.

**Conclusions:** Kostmann syndrome has three main characteristics: severe neutropenia (<0.2 × 10^9^/L), maturation arrest of granulopoiesis at the promyelocyte stage, and death due to infections. HAX1 gene mutations affecting both isoforms A and B are associated with additional neurological symptoms. G-CSF can improve and maintain neutrophil counts, and improve prognosis and quality of life. At present, hematopoietic stem cell transplantation is the only cure.

## Introduction

Severe congenital neutropenia (SCN), also known as Kostmann syndrome, is a rare heterogeneous group of diseases characterized by arrested neutrophil maturation in the bone marrow. SCN caused by HAX1 gene mutation is an autosomal recessive condition that displays recurrent infections of the respiratory tract, skin, and deep tissues from the first few months of life (1–3). Some patients with Kostmann syndrome develop neurological symptoms, including cognitive impairment, neurodegeneration, and epilepsy (4).

The disease should be considered in the presence of an absolute neutrophil count (ANC) in peripheral blood of <500/mm^3^ (5). SCN also shows genetic heterogeneity and demonstrates autosomal dominant, autosomal recessive, X-linked, and sporadic inheritance modes. The autosomal dominant type is the most common, accounting for 60% and is related to heterozygous mutations in the neutrophil elastase (ELANE) gene. Autosomal recessive forms are associated with mutations in the HAX1 and G6PC3 genes (1). Recessive disorders are usually diagnosed in consanguineous populations (3).

We report here the case of a male affected by Kostmann syndrome with intellectual disability and epilepsy and carrier of a mutation in the HAX1 gene. We also reviewed all cases reported in the literature in order to improve our molecular and clinical understanding of this rare disease.

## Case Description

The patient was a 10-year-old Chinese Han male. He had neutropenia accompanied by repeated infections for more than 9 years and fever for 1 week. He was admitted to the Pediatrics Department of the Ninth People's Hospital Shanghai Jiaotong University School of Medicine on March 5th, 2019. When he was 6 months old, his ANC was 0.15 × 10^9^/L by blood routine examination due to respiratory tract infection. According to the family members of the children, the ANC was consistently lower than the lower limit of normal reference range during the following 9 years. Respiratory tract infection, periodontitis, oral ulcer, tonsillitis, and perianal abscess occurred about once a month. Routine anti-infection treatments were used to alleviate the symptoms. Two years ago, the child had a lung abscess. He was diagnosed with epilepsy at the age of 6 years. He was behind his peers in intellectual development and did not go to primary school at the age of 10.

At admission, the father and mother were 53 years old, both in good health, and their marriage was consanguineous. The family history of epilepsy and immunodeficiency was negative. The mother was gravida 5, aborta 1, para 4. Her third offspring, male, died of infection 8 months after birth, but the details are unknown. Her fourth pregnancy was aborted due to social factors. The child reported here was the fifth pregnancy. He had two elder sisters, 31 and 29 years old, both in good health.

The temperature of the patient at admission was 38.4°C, and weight was 34 kg. He was dispirited, with obvious intellectual disability, and with superficial lymphadenopathy. Multiple ulcers could be seen in the lower lip. A mass of 2 × 2 cm could be palpated beside the anus.

**Figure 1:**
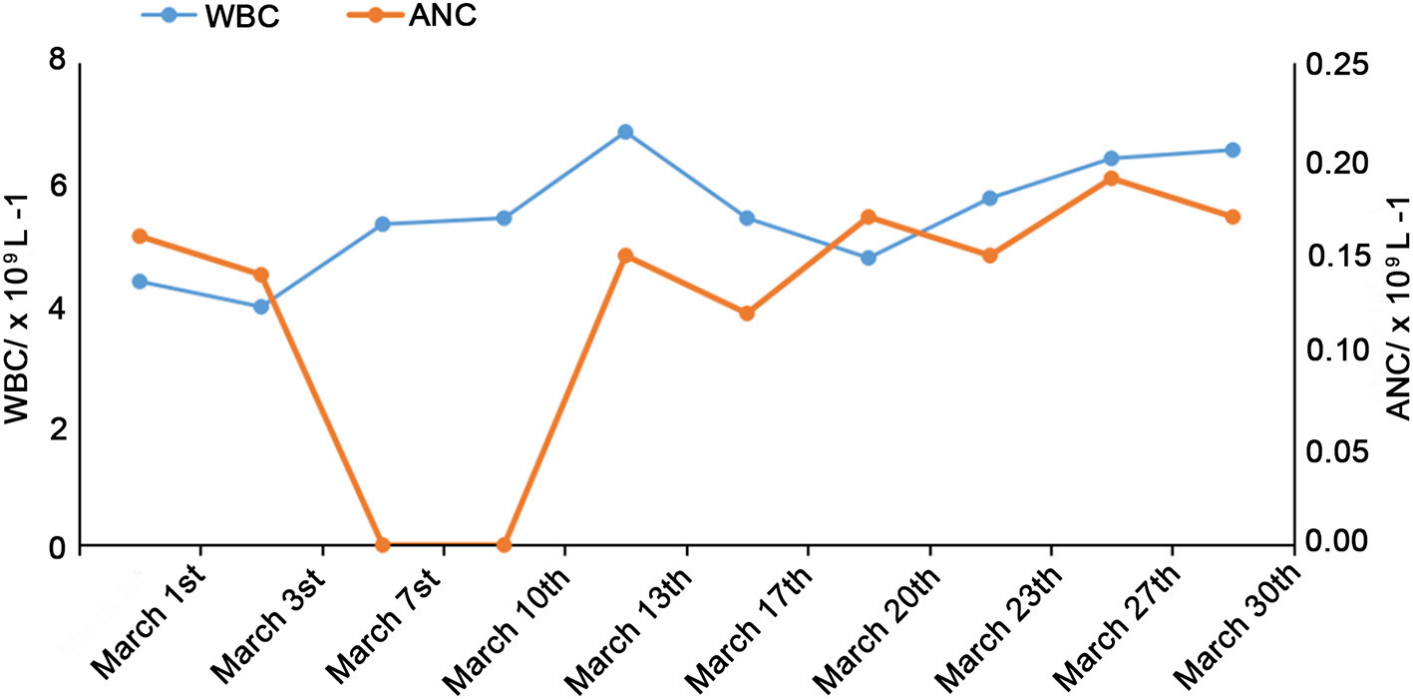
Changes in WBC and ANC over 1 month of follow-up.

**Figure 2:**
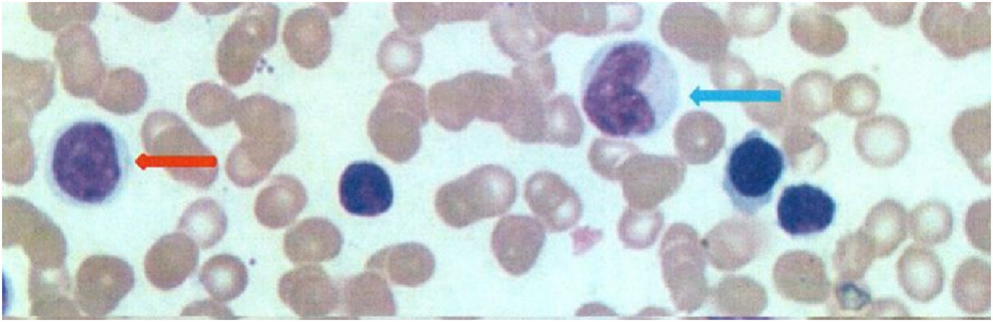
Bone marrow cytology (Rayleigh staining, ×1,000) revealed a granulosis disorder. Red arrow, promyelocytes; blue arrow, promyelocytes.

The white blood cells (WBC, 8–12 × 10^9^/L) and ANC (2.4–4.8 × 10^9^/L) were decreased after the 1-month follow-up, as shown in Figure 1. Bone marrow cytology revealed granulosis disorder, as shown in Figure 2. The immunological and metabolic results were normal.

According to the history of repeated infection, ANC decrease, and the result of bone marrow cell morphology, congenital neutropenia was highly suspected. In order to confirm the diagnosis, with the consent of the family members, sequencing was performed. Whole exon sequencing of the child and parents showed that the child was homozygous for the exon 3 c.430dupG mutation of the HAX1 gene NM-006118 (p.Val144fs) and that both parents carried the same mutation.

## Diagnostic Assessment

At the same time, as anti-infection treatment, the ANC increased to 1.5–2.8 × 10^9^/L after subcutaneous injection of G-CSF (5 μg/kg/d). At the beginning of admission, from March 5th to 9th, azithromycin and cefoxitin were used to manage the infections, while adenosine monophosphate was used as antiviral treatment. WBC and ANC still progressively decreased, as shown in Figure 1, and the fever increased. Oral symptoms were obvious. Cefepime combined with metronidazole was given from March 10th to 14th, and leucoson was used to prevent leukopenia. The fever decreased, the clinical symptoms were relieved, but the increase in ANC was not obvious. From March 15th to 21st, cefoperazone sulbactam and G-CSF were used. On March 22nd, the antibiotics were stopped after clinical symptoms, and laboratory tests were relieved, and the patient was discharged. The patient was followed by phone and WeChat until May 2020. ANC and WBC remained stable with G-CSF, and 3 months elapsed since the last infection occurred.In this case, after the diagnosis, considering the matching and cost of HSCT, bone marrow transplantation was not selected for treatment, but continued to use G-CSF therapy.

## Literature Review

Using “HAX1 gene” and “Kostmann syndrome” as key words, the Chinese and English databases (PubMed, CNKI, Wanfang, and VIP databases) were searched for papers published up to June 15th, 2020. A total of 29 papers about Kostmann's syndrome caused by HAX1 gene mutation were screened. Fourteen papers were excluded because of duplicates, cases without neurological abnormalities, and cases with an incomplete medical history.

The clinical data of 14 children were summarized and analyzed (Table 1). There were 10 males and five females. The family sources were Japanese (*n* = 5), Italian (*n* = 3), Pakistanis (*n* = 2), Turkish (*n* = 1), Kurdish (*n* = 1), Chinese (*n* = 1), and unknown (*n* = 1). The mutations (protein level) were p.Val144fs (*n* = 5), p.Arg86X (*n* = 7), and p.Gln137X (*n* = 1). Eight cases had a nervous system abnormality combined with epilepsy. Twelve patients were treated with G-CSF; eight of them responded well to treatment, with increased WBC and decreased numbers of infections, but the neurological manifestations did not improve. Patient #3 patient received HSCT at the age of 3, but the neurological symptoms worsened after HSCT. At the age of 19, she was unable to have any voluntary, spontaneous motility, but smiles and communicates with her family. After G-CSF treatment, ANC increased slightly in patient #5, but she died of severe sepsis at the age of 6 with neurological degradation and refractory seizures.

**Table 1:** Clinical characteristics of patients with Kostmann syndrome and neurological manifestations caused by HAXI gene mutation.

**#**	**Sex**	**Age at diagnosis**	**Country**	**Mutation (protein level/nucleotide change)**	**Neurological symptoms**	**Seizure**	**Infections**	**Treatment**	**Response to G-CSF**	**References**
1	Male	1.2 years	Turkey	p.Val144fs	Speech retardation	(+)	Gingivitis, stomatitis, recurrent skin infection, pneumonia	G-CSF	(–)	(1)
2	Male	7.2 years	ND	p.Arg86X	IQ:75	(+)	Recurrent upper respiratory tract infection, perianal skin abscess	G-CSF	(–)	(4)
3	Female	5 months	Kurdish	p.Val144fs	Progressive nervous system abnormality	(+)	Skin infection, gingivitis, pneumonia	CSF HSCT	(–)	(11)
4	Male	4 years	Pakistan	p.Val144fs	(+)	(–)	Rhinitis, otitis media, pharyngitis, septic shock	G-CSF	(+)	(11)
5	Female	ND	Pakistan	p.Val144fs	(+)	(+)	ND	G-CSF	(+)	(11)
6	Male	2 months	Japanese	p.Arg86X	Neurodevelopmental delay	(+)	ND	G-CSF	(+)	(12)
7	Female	7 months	Japanese	p.Arg86X	Neurodevelopmental delay	(+)	ND	HSCT	/	(12)
8	Female	4 months	Japanese	p.Arg86X	Neurodevelopmental delay	(+)	ND	G-CSF	(+)	(12)
9	Male	1 years	Japanese	p.Arg86X	Neurodevelopmental delay	(–)	ND	HSCT	/	(13)
10	Male	6 months	Japanese	p.Arg86X	Neurodevelopmental delay	(–)	ND	G-CSF	ND	(13)
11	Male	7 months	Italian	p.Val144fs	Neurodevelopmental delay	(–)	ND	G-CSF	(+)	(8)
12	Male	1.8 years	Italian	p.Gln137X	Neurodevelopmental delay	(–)	ND	G-CSF	(+)	(8)
13	Female	ND	Italian	p.Arg86X	Neurodevelopmental delay	(–)	Skin abscess, otitis media, pneumonia, oral ulcer	G-CSF	(+)	(8)
14	Male	9 years	Chinese	NM-006118: exon3:c.430dupG (p.Val144fs)	Low IQ	(+)	Peristomatitis, oral ulcer, pneumonia, pulmonary abscess, perianal abscess	G-CSF	(+)	This report

*ND, not determined; IQ, intelligence quotient; G-CSF, granulocyte colony-stimulating factor; HSCT, hematopoietic stem cell transplantation*.

## Discussion

HAX1 was originally discovered by Suzuki et al. in 1997 (6, 7). HAX1 is a widely expressed mitochondrial protein that plays an important role in the stabilization of the mitochondrial membrane potential. HAX1 deficiency leads to increased myeloid cell apoptosis (4), decreased expression of anti-apoptotic proteins apoptosis regulator Bcl-2,Bcl2-associated receptor agonist cell death (Bcl-xL) and baculoviral IAP repeat-containing protein 5 (survivin), but enhance expression of Bcl-2-related protein A1 (BFL-1) and induced myeloid leukemia cell differentiation protein Mcl-1 (mcl1/EAT) was detected (3). but HAX1 mutations are relatively rare (8). Among patients with SCN in Middle-Eastern countries, the most common mutation of HAX1 is pW44X (9). Two distinct phenotypes are reported in the literature, which are related to the different isoforms of HAX1. Studies have shown that HAX1 mutations cause neutropenia by affecting isoform A (p.Trp44X, p.Glu59X, p.Glu60fs), while mutations affecting both isoforms A and B (p.Arg86X, p.Gln123fs, p.Val144fs, and p.Gln190X) are associated with additional neurological symptoms, including neurological abnormalities and epilepsy (1, 10). Studies have reported that a homozygous single-nucleotide insertion (position 130-131insA) leading to a premature stop codon (p.Trp44X) was detected (10), but, no mechanism has been reported for other mutation types. Thirteen cases of intellectual disability and/or low IQ were reported in the literature. The mutation sites were p.Val144fs (*n* = 5), p.Arg86X (*n* = 7), and p.Gln137X (*n* = 1) (Table 1).

HAX1-associated SCN is a rare SCN subtype, most commonly seen in patients in Turkey, Kurdistan, and Sweden (11). This syndrome has three main characteristics: severe neutropenia (0.2 × 10^9^/L), maturation arrest of granulopoiesis at the promyelocyte stage, and death due to infections (11). Common initial bacterial infections in patients with Kostmann syndrome include skin and soft tissue abscesses, boils and sores, otitis media, periodontitis, and pneumonia; the mouth and rectum are the most common sites of infection (14). In the case reported here, the patient had recurrent infections for more than 9 years, including peristomatitis, oral ulcer, pneumonia, pulmonary abscess, and perianal abscess. This child also had epilepsy, but the exact link between Kostmann's syndrome and epilepsy remains unclear. However, there is a possible association between epilepsy and immunological abnormalities (15, 16). Carlsson et al. (17) reported neurological abnormalities that could be seen in the autosomal recessive inheritance of HAX1 and G6PC3 mutations. The abnormal mitochondrial function could also be involved. The central nervous system is heavily dependent on oxidative metabolism. In the case of mitochondrial damage with a subsequent decreased energy production, increased oxidative stress, and release of cellular apoptosis factors, the central nervous system might be damaged and develop retardation or epilepsy (4).

Although hematopoietic stem cell transplantation is the only curative therapy for this disease, it can lead to various complications and mortality. Currently, the treatment of Kostmann's syndrome in the literature also refers to the treatment of SCN. It involves the use of G-CSF (3). The overall survival is now estimated to be >80%, including patients with malignancies (18, 19), although about 10% of patients (mainly G-CSF non-responders) still die of severe bacterial infections or sepsis (20). With the use of G-CSF, the mortality of sepsis decreases by 0.9% every year (21). In HAX1-deficient mice, attempts to replace damaged genes with complete ones have achieved some successes (22).

In conclusion, Kostmann syndrome has three main characteristics: severe neutropenia (<0.2 × 10^9^/L), maturation arrest of granulopoiesis at the promyelocyte stage, and death due to infections. HAX1 gene mutations affecting both isoforms A and B are associated with additional neurological symptoms. G-CSF can improve and maintain neutrophil counts and improve prognosis and quality of life. At present, hematopoietic stem cell transplantation is the only cure.

## Data Availability Statement

The original contributions presented in the study are included in the article/Supplementary Materials, further inquiries can be directed to the corresponding author/s.

## Ethics Statement

The studies involving human participants were reviewed and approved by the ethic commitee of the Ninth People's Hospital, Shanghai Jiaotong University School of Medicine. Written informed consent to participate in this study was provided by the participants' legal guardian/next of kin.

## Author Contributions

BL and XZ conceived and supervised the study. WL analyzed data. BL, WL, and XZ wrote the manuscript. WL and XZ made manuscript revisions. All authors contributed to the article and approved the submitted version.

## Conflict of Interest

The authors declare that the research was conducted in the absence of any commercial or financial relationships that could be construed as a potential conflict of interest.
